# How many ways can you die? Multiple biological deaths as a consequence of the multiple concepts of an organism

**DOI:** 10.1007/s11017-022-09583-2

**Published:** 2022-07-20

**Authors:** Piotr Grzegorz Nowak, Adrian Stencel

**Affiliations:** Institute of Philosophy, Jagiellonian University, Grodzka 52, 31-044 Kraków, Poland

**Keywords:** Brain death, Organismal pluralism, Developmental concepts of an organism, Physiological concepts of an organism, Evolutionary concepts of an organism, Soul

## Abstract

According to the mainstream position in the bioethical definition of death debate, death is to be equated with the cessation of an organism. Given such a perspective, some bioethicists uphold the position that brain-dead patients are dead, while others claim that they are alive. Regardless of the specific opinion on the status of brain-dead patients, the mere bioethical concept of death, according to many bioethicists, has the merit of being unanimous and univocal, as well as grounded in biology. In the present article, we challenge such a thesis. We provide evidence that theoretical biology operates with a plurality of equally valid organismic concepts, which imply different conclusions regarding the organismal status of a brain-dead patient. Moreover, the theoretical biology concepts of an organism are very distant from the view on an organism that appears by way of bioethicists theorizing on death. We conclude that if death is to be understood as the cessation of an organism, there is no single correct answer to the question of whether a brain-dead patient is alive or dead.

## Introduction

Since the 1980s, it became a mainstream position in regulatory bioethics to define death “biologically,” meaning “… the permanent cessation of functioning of the organism as a whole” [1] or by a President’s Commission^1^ as “that moment at which the body’s physiological system ceases to constitute an integrated whole” [2, p. 33]. Both we and many bioethicists understand these definitions as simply equating death with the cessation of an organism. For example, Melissa Moschella, in reference to Bernat et al.’s definition, comments that “the early defenders…of neurological criteria for human death take biological integration to imply ontological wholeness (unity) and thus persistence of the human organism” [3]. According to this interpretation, “persistence of an organism” is synonymous with the persistence of “a living organism,” and strictly speaking a dead organism is no longer an organism but rather a former organism. See Table 1 for more citations subscribing to such a view on the debate.

**Table 1:** A sampling quotes identifying death with cessation of an organism

Author	Quote
D. Allan Shewmon	Even if (hypothetically) degree of integration *could* be meaningfully measured, there would be no point along that continuum that could reasonably nonarbitrarily constitute the dividing line between extremely sick, dying organisms, and *just-dead (non-)organisms* [29] (emphasis added)
James L. Bernat	In this article, I offer a refined account of the organism as a whole to more convincingly explain how its cessation spells death [5]
James L. Bernat, Charles M. Culver, Bernard Gert	We define death as the permanent cessation of functioning of the organism as a whole. We do not mean the whole organism, for example, the sum of its tissue and organ parts, but rather the highly complex interaction of its organ subsystems. The organism need not be whole or complete, it may have lost a limb or an organ (such as the spleen), but it still remains an organism [1]
Adam Omelianchuk	Bernat…asserted the loss of the organism itself is what matters. This assertion is deeply metaphysical because human death is linked to human organisms, not some special property of those organisms…. Nor does it permit there to be such things as dead organisms, or at least a dead organism as a whole. It also raises a pressing question: What apart from an organism’s activity indicates that an organism as a whole exists? [6]
Maureen L. Condic	Of course, this [lack of rationality and global, self-integrated organismal function] in itself does not prove that a brain dead body is not a living human organism. More argumentation would be needed in order to show that (1) the capacity for global, self-integrated organismal function is necessary for the persistence of an organism [7]

Besides defining death utilizing the notion of an organism, other proposals have also been present from the very beginning of the debate. For example, Robert Veatch put forward a moral idea, defining the “word death as the name applied to the category of beings who no longer have full moral standing as members of the human community.”[8] (cf. [9]), while Michael Green and Daniel Wikler proposed identifying death with the cessation of personal identity [10]. However, these alternatives were evaluated as too vague to constitute the primary basis for the Uniform Determination of Death Act (UDDA) that was proposed in 1981. President’s Commission, the author of UDDA, noticed that the concepts of moral standing and that of personal identity vary between different people, societies, and cultures [2, p. 39]. Therefore, one can “rely on them only as confirmatory of other views (i.e., biological views) in formulating a definition of death” [2, p. 39], while Bernat et al. states that the concept of “person” “is inherently vague. Death is a biological concept. Thus in a literal sense, death can be applied directly only to biological organisms and not to persons” [1]. This was later echoed in 2008 by the President’s Council on Bioethics statement, according to which there are serious difficulties with John Lizza’s [11, pp. 51–59] ideas, which resemble those of Veatch or Green and Wikler. The members of the Council stated that “one such difficulty is that there is no way to know that the ‘specifically human powers’ are irreversibly gone…” [12, p. 51].

It seems that many bioethicists believe that there is a single univocal and agreed-upon concept of an organism that corresponds to reality and the associated concept of biological death. For example, the conservative President’s Council on Bioethics stated that “death is a single phenomenon marking the end of the life of a biological organism. Death is the definitive end of life and is something more complete and final than the mere loss of ‘personhood’” [12, p. 52]. Meanwhile, the liberal thinker Peter Singer once asked a question that he intended to be a rhetorical one: “Isn’t the distinction between life and death so basic that what counts as dead for a human being also counts as dead for a dog, a parrot, a prawn, an oyster, an oak, or a cabbage?” [13, p. 20]. Quite recently, Andrew Huang and James Bernat, stated that “*death is biologically univocal*” [30] (cf. [2, p. 31– 40; 3; 7; 13, p. 20; 14, pp. 59–85; 15, pp. 1–109; 16–19]), presupposing that all living beings are organisms in the same unified meaning and cease to be organisms in the same sense. These bioethicists and many more have all contributed to the view that we will subsequently call the “biological-bioethical” view on the nature of an organism and on the nature of death. This view will be contrasted with the theories of an organism and the associated concepts of death developed by theoretical biologists and philosophers of biology.

We emphasize in our investigation that, given the plurality of organismic concepts in theoretical biology [20–28], there is no such thing as a univocal biological or easily accessible sense of organism as a concept. Instead, there is a plurality of biological concepts of an organism, which implies that the cessation of an organism is not an idea that can be defined objectively but will rather depend on the concept used in a given situation. Indeed, if we can define an organism in many ways, then the state of “being dead” might vary between them.

Our first aim is to present the “bioethical-biological” concept of death and its implicit presuppositions on the theory of an organism as this concept has been elaborated by different bioethicists engaged in the definition of death debate. We then present the organismal pluralism within theoretical biology and the philosophy of biology. Finally, we reach a conclusion regarding the plurality of biological deaths. We show that there are plenty of biological and “biological-bioethical” concepts of an organism which give different results on the status of brain-dead patients.

## Different variants of “bioethical-biological” view on death and their implicit presuppositions on the theory of an organism

All “bioethical-biological” concepts that are of interest here are elaborated for the sake of determining the status of brain-dead patients. The most classical version proposed by Bernat et al. and the President’s Commission does not imply anything more about an organism than that it is a whole that can exist if, and only if, its subsystems are functionally integrated. Regarding brain-dead patients, the authors of this concept simply stated that they are “merely a group of artificially maintained subsystems since the organism as a whole has ceased to function” [1]. The vagueness of the notions of “integrative functions”, “integrative unity”, and other synonyms were later utilized by Alan Shewmon to argue for the opposite thesis, namely that brain-dead patients are living organisms. Shewmon was the first to operationalize the vague “bioethical-biological” definition of an organism for the sake of resolving the brain-death controversy. According to him, one might be counted as belonging to the class of organisms if one poses a sufficient level of integrative unity, operationalized by two criteria:

CRITERION 1. “Integrative unity” is possessed by a putative organism (i.e., it really is an organism) if the latter possesses at least one emergent, holistic-level property. A property of a composite is defined as “emergent” if it derives from the mutual interaction of the parts,…and as “holistic” if it is not predicable of any part or subset of parts but only of the entire composite.

CRITERION 2. Any body requiring less technological assistance to maintain its vital functions than some other similar body that is nevertheless a living whole must possess at least as much robustness of integrative unity and hence also be a living whole. [4]

In Shewmon’s view, brain-dead patients perform a “litany” of functions that fulfill criterion 1, such as maintaining homeostasis [4, 29]. Moreover, some of them, i.e., those who survive the acute period of spinal shock, fulfill criterion 2, requiring less technical assistance than some patients with high spinal cord transection. Patients in an acute phase of high spinal cord transection suffer from spinal shock and need much more artificial support than stabilized brain-dead patients. For example, they need medication to manage their bradycardia, while some stable brain-dead patients do not require such assistance. Since no one questions that conscious patients with high spinal cord transection are living organisms, the conclusion follows, according to Shewmon, that brain-dead patients are living organisms as well.

Suppose Shewmon’s proposal is to be evaluated as a universal biological definition of an organism. In that case, it has an obvious drawback: due to criterion 2, it presupposes at the outset that we know that some groups of patients count as living organisms [9]. Despite this potential drawback, Shewmon made a significant contribution to the bioethical debate since his analysis compelled bioethicists to say something more about organisms than that they are entities that are functionally integrated. Recently, bioethicists have pursued numerous attempts to clarify the concept of an organism associated with the definition of death debate. For example, Bernat stated that intuitions have an essential role in distinguishing the class of organisms from non-organisms, such as organisms’ parts:

People intuitively grasp that while many parts of the technologically supported brain-dead patient remain alive, the patient has died. The essence of this intuition is the recognition of the fundamental distinction between the life status of an organism’s parts and of its whole. As dramatically shown by the examples of ex vivo cell cultures and tissue and organ transplantation, parts of the human organism can be kept alive for prolonged periods by technology after the organism has died [5].

According to Bernat, intuition plays a crucial role in distinguishing organisms from non-organisms, not only in lay people but also among the scientists engaged in the debate. It is impossible, according to him, to provide a uniform definition of an organism as a whole that is neither too strict nor too broad. He confesses that “the inescapable conclusion is that all members of the immense diversity of life forms cannot be neatly separated into distinct categories delineated by specific criteria that correctly and comprehensively classify them into either living or nonliving categories” [5].

In another recent work coauthored with Andrew Huang, Huang and Bernat notice that there are two incompatible intuitions about human death: on the one hand, we intuit that a human dies in the same sense as other living organisms, but on the other hand, we believe that there is something peculiar in the human way of ceasing to exist, that is, we intuit that patients without any residual consciousness are gone, even though they might be capable of performing many physiological functions, such as spontaneous breathing for example [30].

Huang and Bernat distinguish between a concept and the conception of death. The conception is general and based on a vague notion of an organism as a whole as an integrated, complex entity, possessing some emergent functions, being capable of combatting entropy, and possessing a common ontogenetic. Such a general concept might become more precise in delineating different organisms’ lives and deaths when we identify the “most macroscopic unifying and integrating emergent functions” [30] of a given type of organism as a whole. In this way, it is possible to obtain a conception of an organism as a whole. In humans, Huang and Bernat state that the crucial function is to be identified with neurological control over consciousness and breathing. A supposed upshot is that a brain-dead patient, a patient with a compromised ability to provide neurological control over respiration and consciousness, has ceased to be a human organism, or at least, has ceased to be an organism as a whole.

Huang and Bernat’s concept seems to be best suited to the organismal pluralism of all “bioethical-biological” views. However, even these authors do not acknowledge or discuss their ideas in an organismal pluralism context–this is the general problem with the view. The specific problem with Huang and Bernat’s model is that they seem to simply ignore other “macroscopic unifying and integrating emergent functions” such as the capacity to fight infections or an ability to digest and assimilate resources without which breathing and consciousness fade away, which are for us as much intuitive aspect of human organisms like the one mentioned by them. To be more convincing, Huang and Bernat should analyze intuitions much more closely and justify them as reliable instruments in accessing biological reality. Yet, this seems implausible since it is the culture that mainly shapes the intuitions that Huang and Bernat refer to [31, 32]. In particular, intuitions about the status of brain-dead patients are shaped somewhat by the most famous bioethical texts, including the one authored by Bernat in the 1980s [1]. For this reason, Huang and Bernat’s appeal to intuition is a matter of begging the question: through their recent work utilizing intuitions about organisms, they are trying to defend the concept of death as formulated by Bernat et al. in the 1980s, which itself shaped our intuitions on organisms.

Another idea which helps to pinpoint the “bioethical-biological” concept of an organism comes from Melissa Moschella. She is one of the scholars who have tried to explain the notion of an organism through the Aristotelian-Thomistic theory of the soul:

A putative organism really is an organism if it possesses the *root capacity for self-integration*. Possession of the root capacity for self-integration (of which the soul is the principle) is evidenced [in humans and other sentient animals] by (1) possession of the material basis of the capacity for self-integration—i.e., the capacity for control of respiration and circulation—or (2) possession of the material basis of the capacity for sentience. [18]

Moschella argues that integrated functioning manifested, for example, by the maintenance of homeostasis of the brain-dead body, results from artificial support and does not count as self-integration caused by the soul. She believes that so-called “root capacities” for consciousness and spontaneous breathing are dependent on the brain in adult human beings, so a brain-dead body on artificial support is a dead organism. However, it is hard to understand why (1) and (2) are to be treated as the only proofs of the root capacity for self-integration. Why is the joint function of organs, such as the kidneys, lungs, hypothalamus, posterior pituitary, pancreas, adrenal glands, parathyroid glands, bone, liver, intestines, the bicarbonate buffer system within the extracellular fluid, and the hemoglobin buffer in red blood cells in maintaining homeostasis not perceived as self-integration? [33, 34] Clearly, brain-dead patients would not survive artificial support being turned off, even with all the organs mentioned above undamaged. Still, neither could a patient with a functioning brain and dysfunctional kidneys survive without dialysis or kidney transplantation.

Moreover, even though brain-dead patients cannot regain spontaneous breathing at the current level of the development of medical technologies, they might still have the “root capacity” for spontaneous breathing in Moschella’s sense and possess a human soul. They might be like spinal cord intersection patients where, according to Maureen Condic, “the organizing principle of the body [i.e. the soul] must persist (otherwise the individual would be dead), but the full function of this principle is blocked by an injury-induced material deficiency” [7]. Just as spinal cord intersection patients are living human organisms with the impeded ability to breathe independently, so might be brain-dead patients. They might be capable of recovering spontaneous breath if the physiological obstacles are removed. Currently, scientists can grow mini-brain organoids from stem cells [35]. Perhaps it is physiologically possible to grow a standard size human brain in this way, together with a functioning brainstem and then transplant it into the brain-dead body. Provided that such a brain would be grown from the patient’s cells, genetically it would be the patient’s brain, even though it would be a *tabula rasa* with no mental content. If something like this is physiologically possible, although not yet feasible, it would mean that brain-dead patients *today* have a root capacity for spontaneous respiration, *a fortiori* they have a root capacity for self-integration and a rational human soul.

These conclusions, which are undesired by Moschella, could be avoided if we look at the other model of organism unity enshrined in another of her works. According to this view, each organism exists so long as its master part persists. Such a part is understood as “the vital, essential part that has the biological function of controlling all of the organism’s parts, directly or indirectly” [3], (cf. [36]). The view according to which a master part is a sine qua non condition for the existence of each organism is reasonable, since as Hoffman and Rosenkrantz argue, all known organisms have a master part [37]. According to Moschella, it is beyond controversy that the central nervous system constitutes a master part of adult human organisms. Therefore, humans without functioning brains no longer form human organisms.

A structurally similar approach to Moschella’s first idea of defining an organism in terms of the function of the “self” was adopted even earlier by the President’s Council of Bioethics. It was enshrined in position no. 2 of the “Controversies in Determination of Death” report. The Council pointed out that the concept of death developed in the 1980s by Bernat et al. and the President’s Commission was right in perceiving an organism as *a whole*. However, it was wrong to interpret an organism’s wholeness as functional or somatic integration [12, pp. 59–60]. Instead, the President’s Commission proposed to define “organisms as a whole” as entities, capable of performing “the work of self-preservation, achieved through the organism’s need-driven commerce with the surrounding world” [12]. In turn, the capability to perform this vital work was interpreted as being dependent on three “fundamental capacities”:

1. Openness to the world, that is, receptivity to stimuli and signals from the surrounding environment. 2. The ability to act upon the world to obtain selectively what it needs. 3. The basic felt need that drives the organism to act as it must, to obtain what it needs and what its openness reveals to be available. [12, p. 61]

Many commentators have already noted that this concept of an “organism as a whole” is more unclear, underspecified, and nonscientific than the view formulated in 1981 by Bernat et al. and the President’s Commission [17; 34; 38; pp. 72–75; 39]. The most severe problem with this definition of an “organism as a whole” is that it is circular: it defines an organism as a whole in terms of self-preservation. However, what is at stake is indeed the self-preservation of *an organism as a whole*, so we need first to know what an “organism as a whole” is before considering whether it can perform “the vital work” of preserving itself. However, the President’s Commission does not explain their concept of an “organism as a whole” further.

Recently, Adam Omelianchuk proposed a strategy to address this gap [6]. According to him, the work of self-preservation should be interpreted as a “second-order capacity (viz. a capacity for having a capacity) for self-movement towards species-specific ends” [6]. Note that the notion of “second-order capacity” is quite similar to Moschella’s idea of “root capacity.”

In our view, such a defense of the President’s Council idea is problematic due to several reasons. First, the idea that there are species-specific ends is based on an Aristotelian metaphysics which, with its final causes, soul, entelechies, and so on, is foreign to contemporary natural science, [cf. 34]. Second, even if we agree for the sake of argument that there are final causes, and so are species-specific ends, moreover, if we agree that the distinctively human end is that of rational thought and action, it is hard to understand why Omelianchuk upholds that anencephalic newborns and persistent vegetative patients (PVS) are alive while brain-dead patients are dead. What is the difference between these groups of patients regarding the second-order capacity for rational thought and action? To justify this distinction, Omelianchuk states that anencephaly and PVS are only disorders that impede the capacity for rational thought and action. At the same time, brain death is more than an impediment. It is the destruction of the second-order capacity for the achievement of human ends.

Yet, just as there is currently no therapy that could help the brain-dead regain consciousness, there is no treatment that might help anencephalic newborns develop consciousness either. Thus, it is hard to understand why anencephaly impedes the second-order capacity for rationality while brain death destroys it. Moreover, we can recall the case of brains grown from stem cells discussed in the context of Moschella’s view. If it is physiologically possible to grow brains from a brain-dead patient’s steam cells, perhaps these patients have not lost the second-order capacity for rational thought and action. If this is the case, brain death is only an impediment to the second-order capacity of being conscious.

In terms of its scientific background, the most promising “bioethical-biological” concept of an organism utilizes the modern scientific notions of homeostasis and entropy. Julius Korein was the first in the context of the definition of death debate to define organisms as open systems that tend to minimalize their own entropy and maximize their negentropy at the cost of increasing entropy in the environment [40]. Much more recently, Michael Nair-Collins defined organisms in the following manner:

Living organisms are localized pockets of anti-entropy, achieved by mutually interdependent functional structures jointly maintaining internal equilibrium, or homeostasis of the extracellular fluid, a necessary condition for all organismic function, while resisting chemical and thermal equilibrium with the external environment. Second, living organisms are a social collective, consisting of trillions of cells working together to actively maintain their environment within conditions suitable for their continued functioning and existence. [34]

Given such a definition of an organism, Nair-Collins noticed that entropy and homeostasis are inversely related and identified death with “the irreversible cessation of the organismic capacity to maintain homeostasis of the extracellular fluid and thereby resist entropy” [34]. While Nair-Collins believes that brain death does not constitute the death of a human organism, Korein holds the adverse opinion. According to him, it was impossible to maintain the functioning of a mature brain-dead human body for a period longer than a week. We know today that this statement has proven to be false [41].

The entropic-homeostatic concept of an organism has certain merits over the classic “biological-bioethical” view given its precise nature. “Entropy” is not a nebulous concept like “integrated functioning,” and its change is formally operationalized by scientists as the measurement of the dispersion of energy at a stated temperature [42]. According to Nair-Collins, the concept has the essential merit of being a “part of a coherent, unified story of the world and our place in it, drawing on a well unified ontology within a mechanistic explanatory framework” [34]. Perhaps it is the part of the “unified story of the world,” yet we are afraid that it might be neither a story of “*our* place” in the world nor even the story of organisms, but the more general story of living matter.

The entropic-homeostatic concept tells us only that if we want to know whether x (still) constitutes a living organism, we should consider whether x might be capable of maintaining homeostasis and thereby resisting entropy. Yet, it gives us no clues as to how we might solve the crucial issue in the philosophy of biology of recognizing the borders between different organisms, and it provides no instruments for differentiating organisms from their parts [43]. Given this theory, we have no advice on where to find the borders of some person’s organism. Suppose we consider the borders of Adam’s organism. Are these borders coextensive with the commonsense notion of Adam’s body? Or perhaps the boundaries coincide with the commonsense idea of Adam’s body plus his microbiota? A different option is to identify an individual organism as constituted by the objects we term the commonsense notion of Adam and Bill’s bodies. Yet another idea is that the individual organism might be formed solely by Adam’s kidneys. Biological concepts, such as the immunological, or zygotic concepts, can provide answers to such questions (see the next section), while the entropic-homeostatic view is incapable of serving this purpose [cf. 43]. This concept is perhaps a fragment of the true story of the world. However, it is not a story about individual organisms but rather one about the difference between living and inanimate material. Since it cannot facilitate the distinction between an organism and its parts, it cannot answer the question of whether a brain-dead body constitutes an individual organism or perhaps only a part of an organism, albeit a large one [cf. 3, 18, 19]. The concept can only confirm the obvious truth, namely that brain dead patients on artificial support are part of the living world as opposed to inanimate material such as rocks.

It seems that while looking for a scientific theory of the death of an organism, followers of the homeostatic-entropic concept have focused on the wrong part of science. To see that this is the case, let us notice that the idea of engaging the concept of entropy in theoretical biology investigations originally comes from Erwin Schrödinger’s book “What is life?”. The question settled in the title of the book was answered by Schrödinger in the following way:

When is a piece of matter said to be alive? When it goes on ‘doing something’, moving, exchanging material with its environment, and so forth, and that for a much longer period than we would expect an inanimate piece of matter to ‘keep going’ under similar circumstances. When a system that is not alive is isolated or placed in a uniform environment, all motion usually comes to a standstill very soon as a result of various kinds of friction; differences of electric or chemical potential are equalized, substances which tend to form a chemical compound do so, temperature becomes uniform by heat conduction. [44, p. 69]

Although Schrödinger writes in his book that organisms are alive, meaning that they avoid decay through exchanging material with their environment, minimalizing their inner entropy through metabolism, we believe that organisms are only particular examples of living systems. In addition, parts of organisms such as the above-mentioned kidneys or even cells are alive in Schrödinger’s sense. Moreover, the whole ecosystem of Earth, as opposed to Mars, might be perceived as alive, taking into account the quotation from Schrödinger. Schrödinger aimed to grasp the general nature of life instead of providing a comprehensive theory of an organism [45].

The above discussion on the entropic criterion reinforces our point that if the discussion in bioethics on a definition of death is to be interpreted as the discussion on the requirements an individual organism must fulfill to go out of existence, it needs more reference to contemporary work in the philosophy of biology. This is because it conflates two questions: (i) how to define organisms [20–25, 27, 28, 46]; and (ii) how to define life [47–49]. The two often seem to be confused. From the point of view of the problem discussed in this paper, only the first question seems to be relevant because brain death obviously does not transform organisms into non-living matter. No one would argue that cells and the human body always become non-living matter after brain death–certainly not in the time frame that bioethicists are interested in. The question is to realize whether the link between elements of the human organism is broken to the extent that it ceases to exist. Thus, to answer that type of question we have to understand what an organism is, rather than what is life. In other words, living matter might still be part of something that used to be an organism. Thus, figuring out whether something is alive does not set up the debate as to whether it is an organism. Ant societies are undoubtedly alive, but this does not help us to realize whether they are individuals or a group of individuals [50, 51] and that is why the two questions are separate in the philosophy of biology.

## The plurality of concepts of an organism in theoretical biology

The status and meaning of the concept of an organism is one of the greatest issues to have been raised in the philosophy of biology in recent years. Despite the fact that a bioethical consensus on the definition of death was established in the 1980s, the emphasis in theoretical biology throughout the 20th century was placed on genes and other sub-elements of cells rather than on the concept of an organism [52]. This was partially caused by the fact that during this time, biologists had unraveled the mystery of DNA and learned a lot about the molecular mechanisms of many traits. Thus, the very concept of an organism in theoretical biology was somehow put aside. However, this has changed recently as an increased focus has been placed on understanding “organisms as a whole,” a concept which is also crucial for those engaged in the definition of death debate [cf. e.g., 1; 2, pp. 1–75; 5; 6; 12, p. 1–121; 30]. So, what is an organism from the philosophy of biology point of view? What conditions does something have to fulfill to be considered an organism? Can we unequivocally put forward conditions to call something an organism? Many researchers have tried to tackle this issue and alternative approaches to the concept of organisms have been proposed over the years. For instance, Ellen Clarke [28] counted at least thirteen concepts of an organism in use in 2010. Given the explosion of interest in the topic in recent years, with many papers published [23, 25, 51, 53–55], special issues edited, and conferences organized on this subject, one might expect that the number of concepts has at least doubled.

Let us present a few of the most popular concepts found in philosophy and biology that will fuel our further discussion. We selected a number of concepts from different fields where scientists pursue different goals (e.g., development, physiology, evolutionary biology), in order to show the diversity of concepts that exist in biology.

The most classic concept of the organism in theoretical biology is called the developmental concept of an organism. The concept has been around for about 170 years and was put forward even before the publication of Darwin’s famous work. It was T.H. Huxley who wrote that: “the individual animal is the sum of the phenomena presented by a single life: in other words, it is, all those animal forms which proceed from a single egg taken together” [56]. The developmental concept of an organism is an enduring one and one of the most popular ways of defining the organism by people working on development. For instance, Gilbert et al. defined it in the following way: “the individual animal proposed here is understood to be that which proceeds from ovum to ovum” [22]. Similarly, Moore et al. stated that “human development begins at fertilization when an oocyte (ovum) from a female is fertilized by a sperm (spermatozoon) from a male. Development involves many changes that transform a single cell, the zygote, into a multicellular human being” [57, p. 1].

Thus, this concept emphasized development as the process that marks the difference between two individuals. Here, organisms come from a fertilized egg and consist of all the cells that make up its body, like muscle cells, nerve cells, or the cells that build the digestive tract. All those cells coming from the fertilized egg constitute a developmental organism until the next process of fertilization, which marks the emergence of another individual. The concept seems to be quite correct: you and your friends are different individuals because you all developed from different fertilized eggs. However, in other cases, it generates quite strange, non-intuitive individualization. For instance, Janzen [58] argued in a famous paper that if an organism reproduced from an unfertilized egg, as is the case with many species (dandelions, fungus, aphids), then this should not be considered as a process of generating a new organism, but only growth because there is a lack of a sexual event of fertilization that marks the difference between two individuals. Thus, all aphids that grow in a given meadow from an unfertilized egg should be considered one organism, albeit one that is physically disconnected across the meadow. This would mean that twins from a zygote that at some point has undergone mitotic division would also be considered a single individual because there was no sexual event of fertilization to mark the differences between them. Note that bioethicists engaged in the brain death debate sometimes take it for granted that twins cannot be perceived as a single organism [3], which seems to be a proper approach for carrying on bioethical considerations, as twins are so disintegrated in many dimensions that it is justified to treat them as separate units for bioethical investigations.

If we try to look at this concept from the perspective of the bioethical debate, then we realize that it implies a certain way of thinking about the status of brain-dead patients. This is mainly because, since all the elements of such patients are derived from a fertilized egg, they should be considered developmental organisms. Therefore, we should consider it as an organism, even if it starts to decay. Furthermore, even if the body of an organism is turned into dust, it can be still alive if its twin is around–since they constitute a single organism coming from a fertilized egg.

The peculiar consequences of this concept that link such different, physically disconnected elements into a single organism have led scholars to propose an alternative that emphasizes functional integration, one that we can call the functional developmental concept of organisms [25]. The main conceptual problem of the classical developmental concept of an organism was the presupposition that all life forms coming from the fertilized eggs are functionally integrated. It might not always be the case, however. If we explicitly assume that there must be some sort of functional integration among elements that come from a fertilized egg, then this excludes cases like twins–they are rarely functionally integrated in any way. Furthermore, it excludes cases of considering the decaying body as an organism as well, as such an aggregation of cells is not functionally integrated. At the same time, this would deliver a different verdict on the status of brain-dead patients. Suppose they are functionally integrated due to some medical equipment provided by doctors, as is often the case with intensive care units. In that case, they should no longer be considered organisms–because those elements do not come from fertilized eggs, while this kind of origin is a sine qua non condition for the existence of an individual organism, given all variants of the developmental concept of an organism. Therefore, conscious patients who are dependent on pacemakers or transplanted organs in their integrated functioning are also not organisms, but rather something akin to cyborgs.

The functional developmental concept of an organism operates with the notion of functional integration, one which is familiar in the bioethical definition of death discourse. For example, Bernat et al. define an organism functioning as a whole to be an entity whose “spontaneous and innate activities” are carried out by “the integration of all or most of its subsystems,” and is capable of “at least limited response to the environment” [1]. Yet, despite several attempts to operationalize it, the notion of “integrated functioning” (see the previous section for discussion), remains “undefined and vague in the views of those who attempt to define death” [59]. The functional developmental concept of an organism is not different here in this matter–it understands the term intuitively and without elaborating it.

Is the notion of functional integration always as unspecified within theoretical biology as in the case of the developmental concept of an organism? To answer this question, we will discuss another concept that fundamentally relies on functional relations—namely the physiological concept of an organism [e.g., 22, 23, 27, 60]. According to the physiological conception of individuality, if a group of entities engages in a significant amount of physiological interactions with one another, then the group of entities will be considered a physiological individual. In contrast to the developmental concept of an organism, this view does not require that elements making up an organism go through a certain type of development. The origin of the elements of the organism is not very important, but what matters is the existence of certain functional relations. Indeed, the physiological concept of the organism focuses rather on the certain mechanisms of cohesion that make a group of elements a single unit, rather than a group of single units.

As good as it sounds, this idea is approached by scholars in different ways [e.g., 23, 60, 61], and one approach that seems to provide good criteria for the fuzzy term of “functional integrations” is the immunological concept of an organism [23, 61, 62 pp. 239–269]. This approach focuses on immunological properties as the main drivers responsible for setting the boundaries of physiological individuality. Traditionally, the immunological conception of individuality has assumed that the immunological system acts as a gatekeeper that determines the boundaries between the self and the non-self by triggering an immune response in order to eliminate any possible intruders. Self-elements are those that do not trigger an immune response while non-self-ones are those that do. Furthermore, the elements that belong to the self are generally considered those that come from “inside” (i.e., from the zygote) [23]. Thus, the distinction between the self and non-self is quite obvious.

More recently, the immunological view of individuality has emphasized that immune responses are more diverse, and the boundaries set by the immunological view are considerably more dynamic [see 23, 61]. Firstly, constituents that come from the zygote can trigger an immune response even in healthy people [63, 64]. Secondly, there are elements that do not come from the zygote but are tolerated by the immune system, such as symbiotic microbes [65]. This leads to the idea that immunological individuality should be conceived in a more dynamic fashion [23, 61]. This implies that in the context of this concept a given element of the organism (like a nervous cell) might one day be considered part of an organism because it is tolerated by the immune system. At the same time, a few months later, it might be excluded as an element of the organism if it is not tolerated by the immune system anymore. This might transpire because, for instance, a disease such as cancer [66] changes the immunological properties of the immunological system as it does not tolerate some cells anymore. The immunological system defines the boundaries of the organism, and as long as there is an immunological system, as long as an organism exists, its constituents might change dynamically. In other words, whether some elements are part of a given individual should not be based on their origin (from the zygote vs outside), but rather the emphasis should be placed on their tolerance. If they are tolerated by the immune system, then they are part of the organism. This was summarized nicely by Pradeu:

Immunological criterion suggests that any entity which interacts regularly with the immune system and is not eliminated by it is part of the physiological individual. In other words, the physiological individual, immunologically, is the unit made of the association of a host and many microbes (those that are tolerated by the immune system. [23]

If we consider the bioethical definition of death debate from the point of view of the immunological concept of an organism there are several interesting takeaways. First, it seems that participants in the bioethical definition of death debate do not account for the immunological concept in their investigations. Their conclusions quite often clash with the conclusions of proponents of the immunological view of an organism. For example, in contrast with the above quotation from Pradeu, Melissa Moschella writes that:

Both termite and protozoans live within an enclosed membrane in a complex symbiotic relationship, dependent upon each other for survival, functioning in a coordinated manner in the service of a larger whole. Yet they do not constitute a single organism. The protozoans are not parts of the termite. Rather, each protozoan is itself an organism, distinct from the termite. Any plausible account of organismal unity must be fine-grained enough to explain cases like this one. [19 cf. 3, 36]

A second important thing to note is that, given the immunological view, brain-dead patients are living organisms. That is because they are capable of “fighting of infections and foreign bodies through interactions among the immune system, lymphatics, bone marrow, and microvasculature” and the “development of a febrile response to infection” [4]. Finally, the third factor which might have some influence in bioethical contexts other than in the definition of death debate, is the fact that transplanted organs are not genuine body parts of the recipient since they are not recognized as such by their immunological systems [67]. It is an interesting upshot since it raises questions regarding the content of the moral right to bodily integrity. Do we always use the phrase “human body” in a manner synonymous with the biological meaning of a “human organism”? For the moment, we place this fascinating question to one side.

We can now move to the evolutionary concept of an organism–one which is supposed to capture what constitutes an organism from the evolutionary perspective. In other words, it is supposed to state when a given individual is a unit that undergoes evolution by natural selection (ENS). Classically, as indicated by Lewontin [68], for a population to undergo ENS its members must be characterized by variance, fitness differences, and heritability. This classic formula has recently been elaborated in detail [69, 70]. According to this elaborated view, evolutionary individuals (Darwinian individuals) are units that are capable of reproduction. In other words, an evolutionary individual is every unit that is capable of producing offspring. This is an important factor since reproducers are causally responsible for parent–offspring similarity (fulfilling the heritability criterion mentioned above). Thus, if you have a group of reproducers that vary in some traits, those traits influence their fitness (number of offspring), and those traits are heritable, then you can expect that the population will undergo natural selection because some reproducers will produce more offspring than others and, as a result, their frequency will change in a population.

Very diverse types of reproducers exist in nature [71, pp. 87–109, 72] and three paradigm cases of reproducers can be distinguished. The first is a scaffolded reproducer, and they are characterized by the fact that their reproduction is entirely dependent on external machinery. For instance, viruses belong to this category because they use cells to reproduce. The second category consists of simple reproducers. Simple reproducers only need external resources to initiate reproduction, (e.g., a bacterial cell). The third category constitutes collective reproducers which are built of simple reproducers. In other words, a collective reproducer is an entity that can reproduce itself, but which is also built of elements that can reproduce themselves. An example would be multicellular individuals built of eukaryotic cells.

The above concept of individuality based on the theory of evolution is not the only concept present in the literature. There are other concepts that use the theory of evolution to single out organisms from the environment to a greater or lesser extent. One can list here the replicator-vehicle/interactor framework [73, 74], the concept of Organismality [72], “It’s a Song Not a Singer” [75], the concept of Stability of Traits [26] or the the concept of Unity of Purpose [76, pp.43–72]. The latter frame organisms in terms of agency and will be discussed here. Agency is a very important metaphor in considerations of the theory of evolution. It treats organisms as agents similar to human beings. Mainly, as units that have traits that help them pursue some goals. For instance, it is uncontroversial to say that a peacock’s tail has evolved to attract mates. Indeed, we can easily ascribe functions to the majority of biological traits and show that the function of those traits is to increase the fitness of their bearers. This led Okasha [76, pp. 4372] to define organisms along those lines. According to his view, a given unit is mainly an evolutionary agent–an organism from the evolutionary perspective–if it possesses the “unity-of-purpose.” In other words, the different traits of an organism have evolved because of their contribution to the same goal–enhancing bearer fitness.

Even though it might seem that evolutionary agents and Darwinian individuals are quite the same, once we delve into the details it is not so simple. A good case to illustrate this would be the part of noncoding-DNA that people sometimes term “junk DNA”. This is a part of DNA that does not perform any function–although some junk DNA might turn out to be functional in future studies–however, it is replicated with the rest of the genome during cell divisions [77]. If one considers these to be Darwinian individuals, then one might come to the conclusion that junk DNA is part of its bearer’s individuality–because it is replicated together with the rest of the genome, it constitutes part of the genome. For instance, when humans reproduce, we transfer that DNA with all our other genes. At the same time, junk DNA does not perform any function that benefits the fitness of the bearer; thus, it would be hard to say that it contributes to the unity-of-purpose of a human being as it does not benefit the bearer’s fitness. Indeed, it seems the junk DNA has not evolved to enhance the fitness of the organism and thus it would be uncontroversial to say that it does not constitute part of a human organism if the organism is understood as an evolutionary agent.

Two approaches based on evolutionary considerations will differ as well in their verdict about the status of brain-dead patients [78]. If we follow the Godfrey-Smith approach pointed out above, after brain death, the nature of the reproducer changes. The collective reproducer (i.e., a human being) becomes a scaffolded reproducer, as its reproduction becomes dependent on medical equipment and other reproducers (i.e., doctors in charge of the equipment)–so the brain-dead patients eventually resembles cellular organelles like mitochondria or chloroplast that used to be free-living entities, but for now can only reproduce with the assistance of the cellular machinery of eukaryotic cells. Alternatively, someone might take a different route and argue that being unable to reproduce does not rule out the status of “evolutionary individual.” One can zoom out and argue that individuals that cannot reproduce are still evolutionary individuals if they are part of an evolving population. After all, nature is full of individuals that are unable to reproduce for one reason or another, like mules. So as long as the unit is part of an evolving population, it is an evolutionary individual. This point was elaborated in detail by Chodasewicz [79].

The agency view of the organism would lead us to a different conclusion. This view says that a given unit is an evolutionary agent if its different traits have evolved because of their contribution to the same goal–enhancing its bearer’s fitness. At first glance, it might seem that this property is still present in brain-dead patients because several capacities which promote an organism’s fitness are present in brain-dead patients. Besides the capability of generating an immune response to infections, they are also capable of: maintaining homeostasis; the elimination, detoxication, and recycling of cellular wastes; maintaining energy balance and body temperature; wound healing; cardiovascular and hormonal stress responses to unanesthetized incision; sexual maturation; and proportional growth [4]. Moreover, the undamaged spinal cord in brain-dead patients is capable of performing some integrative functions and even of primitive sensorimotor learning, which might manifest itself in an extreme form by means of the Lazarus sign [4]. Of course, all those works only contribute to enhancing fitness if they are assisted with medical equipment, which triggers another issue. However, unity-of-purpose is obtained only if *different traits have evolved* to obtain the same goal. The problem is that medical equipment is not a property that has evolved to enhance fitness; it is a tool made by humans, helpful for enhancing fitness, as is the case with many other tools, but not a phenotype trait that has evolved over thousands of generations in the same way that the eye or the brain have done. Therefore, if the organism must rely on it, it seems it should not be considered an evolutionary agent, because it cannot obtain its goal solely on the basis of the traits that have evolved to do so. However, it seems the same conclusion should follow for patients with functioning brains that rely on their functioning and reproduction on any kind of artificial technology.

This, of course, is only the consequence if we assume that absolute unity-of-purpose is required. In fact, the-unity-of purpose quite often breaks down due to the fact that conflicts within organisms emerge. As a result, Okasha [76, pp. 43–72] argued that we should accept that sometimes unity-of-purpose breaks down because some traits evolve in a neutral manner or to benefit some element below the organism level, like genes for example. Furthermore, these biases from the unity-of-purpose seems to be widespread in nature [80, pp. 1–18]. So, if unity-of-purpose breaks down at times and does not exclude something as an evolutionary agent, it might sometimes be the case that the evolutionary agent is sustained by “traits” that have not evolved for this reason. Indeed, perhaps we can say that sometimes some traits did not evolve to sustain the unity of purpose, but they do so currently, like artificial technology. We think it is a justified position and interesting issue to be explored, but of course, this requires being discussed in the framework of Okasha, and we do not have time for this here. Yet if this is so, then people that are supported by medical equipment, including those with destroyed brains, would be considered evolutionary agents.

To sum up, in biology and philosophy of biology we do not have a universal concept of an organism. The concept of an organism is equivocal and unclear–rather it may be more accurate to say that multiple concepts coexist. The same is true for the concept of death, which might be understood differently depending on the concept of an organism adopted. Furthermore, it is sometimes not even clear how we should understand the concept of death if we stick to a single concept, which comes from the lack of proper analysis of the problem. We have presented how death should be understood in the context of different concepts, but note that as the topic is unexplored, we might be wrong in many places. Our aim was not to provide a definitive answer, but rather to show that neither the notion of an organism nor death is clear. All this shows that philosophy of biology and biology, just like bioethics, cannot currently deliver either a universal concept of an organism or one of death. However, this does not necessarily mean that the concept of an organism is waiting to be developed. Perhaps pluralism is to be expected? This issue and other related ones will be discussed in the next section.

## Four remedies for the gap between bioethics and theoretical biology

As evidenced in the prior section, there are multiple concepts of an organism in the philosophy of biology that give a different verdict about death. There are also numerous versions of the “bioethical-biological” concept of death and an organism (see above). Besides the notion of functional integration and its synonyms, the biological and “bioethical-bioethical” theorizing on organisms differ quite substantially. Furthermore, theoretical biologists are relatively aware of the plurality of the biological concepts of an organism and the fact that it might be hard to deliver a universal concept of the organism. Meanwhile, bioethicists engaged in the definition of death debate seem to avoid taking this into account while developing their arguments. An upshot of this is that there is a considerable gap between the investigations of theoretical biology on the nature of organisms and the bioethical understanding of the issue.

In our view, bioethicists engaged within the definition of death debate in the present situation might consider four options: (1) they might try to show that pluralism is simply wrong and that there is a universal concept of the organism and perhaps even one of the concepts developed by them can serve such a role; (2) they might accept pluralism about the concept of the organism and subscribe to one of the actual biological views as described herein, and accept their conclusions on the status of brain-dead patients; (3) they might take organismal pluralism at face value and defend their concept of an organism as one of many views of an organism which is valid within some subdiscipline of natural sciences, namely within some subcategory of medical sciences; finally (4) they might claim that the death of an organism is a phenomenon distinct from the cessation of an organism. The rest of this section discusses these four options. Whatever bioethicists choose, they can no longer continue with a strategy of “isolation” from contemporary ideas about organisms. We think as well that biologists and philosophers of biology can gain much from the debate about organisms in bioethics, as some of the concepts found here, such as the one developed by Moschella [3], differ substantially from biology and so might provide interesting insights. This cross-fertilization can certainly benefit both, but we will not discuss the potential influence of bioethical concepts on the philosophy of biology as it is not the aim of the paper.

The option (1) of the four mentioned above seems the one which is the most implausible. Its adoption would require the refutation of the dominant position of pluralism in the philosophy of biology [20, 22–25, 27, 28, 46]. Furthermore, it would require developing a concept of an organism that would be universal or showing that one of the concepts found in bioethics or biology can serve such a role. While this might be possible, it would require a lot of effort, and thus if one wants to claim the superiority of a given concept of an organism, one would have to show that organismal pluralism can be replaced by monism. This is a difficult and ambitious project to pursue and to date there have been very few attempts. One well-known example is the work of Ellen Clarke [53] who tried to provide such an account by arguing that all concepts can be reduced to a single one. In such a view, there are a “set of conditions,” meaning, in her view, the existence of “policing” and “demarcation” mechanisms, that, if fulfilled, single out organisms. However, those conditions are realized by different mechanisms in different lineages due to the different evolutionary history of species. Thus, multiple concepts of an organism exist, because people confuse “conditions” with the mechanisms through which they are realized. Once the distinction is made, we can turn pluralism into monism. There exist universal conditions for distinguishing organisms that are realized by various biological mechanisms.

Technically, her idea seems very convincing. However, philosophically speaking, the idea is very problematic. How does one single out conditions? What makes one feel that a given set of conditions is the most appropriate? Clarke [53] believes that evolutionary theory unifies biology, and therefore conditions should be based on it. Sadly, there are two problems with this position. Firstly, it is unclear whether bioethicists would be happy with the idea that their discussion of death should be rooted in the theory of evolution since this theory also does not provide a unanimous verdict on the status of brain-dead patients (see above section). If they are not, they would have to show that some of the conditions they prefer are superior to evolutionary ones. Secondly, many authors have suggested that different conditions are equal to each other [23, 25, 81], so one would have to provide a philosophical argument against pluralism. Of course, one can try to build such an argument, for instance following Clarke [53] and argue that for epistemological reasons (like counting organisms) such a concept is necessary. Furthermore, one might try to refer to more ontological arguments, for instance, to Ockham’s Razor, which requires that we do not multiply entities without necessity–a reference to this principle is very popular in the philosophy of biology [82, pp. 153–239]. However, thus far such a concept and the philosophical arguments to back it up have not been put forward in such a compelling way to convince the majority. As a result, pluralism is the dominant position [23, 25, 51, 53–55] toward which we are sympathetic as well. To sum up, if bioethics wants to defend the monistic view of organisms, then this is a legitimate position, but numerous philosophical investigations are needed to develop and defend it. Indeed, monism about the concept of an organism cannot be considered as something a priori, as it is commonly done in consideration of bioethics, as we have shown in the second section.

In the last paragraphs we have shown that monism about the concept of an organism requires a lot of work to be defended and not everyone would be satisfied with the solution, even if this were possible. Of course, bioethicists can also accept pluralism about the concept of the organism and follow one of two paths. They might choose option (2) or (3) from these listed above. Before outlining these paths, a word is required here concerning the notion of pluralism itself. Pluralism about the concept of an organism is something that seems to be a weak position. There are multiple concepts of an organism, so we have to accept them. However, some scholars have tried to provide ontological justification for pluralism, developing it into a mature philosophical framework. There are many approaches, but two are quite popular and will be outlined below. Both rest on the idea that the concepts of an organism depend on the research aim, with the first explaining it in terms of pragmatism [21, 25, 81], the other in terms of process ontology [83, 84].

Let us start from the pragmatic justification for pluralism concerning the concept of an organism. The idea here is that the concept of an organism has a special role in biology. It is a tool that is supposed to help solve scientific problems. Mainly, if scientists have a problem to solve, a concept of an organism is developed to fit the needs of scientists that pursue the research tasks. The way they define organisms reflects the goal they want to achieve. In other words, the concept is goal-oriented and emerges within the context of given research; it is rooted within the theoretical and empirical basis of a given scientific discipline [21, 25, 81]. Mainly, different concepts of an organism exist because researchers in different fields of the biological sciences are interested in solving different problems, and thus they use different concepts of an organism to pursue those different desires. This was nicely summarized by Wolfe, who wrote that, “the organism is neither a *discovery* like the circulation of the blood or the glycogenic function of the liver, nor a particular biological *theory* like epigenesis or preformationism. It is rather a *concept* which plays a series of roles–sometimes overt, sometimes masked–throughout the history of biology, and frequently in very normative ways, also shifting between the biological and the social” [81].

The other approach depends on the ontology of the process [83, 84]. The idea here is that organisms are not some sort of “things” or philosophically speaking, “substances,” but rather some sort of processes that undergo constant changes. They integrate elements of the environment, their parts (like organs) wear out and are replaced by other elements (cell with another cell) and some of them even undergo drastic changes during development. Just think about the tremendous change human zygote or insect eggs undergo when they are transformed into adults. They constitute the flow of living matter, and we only capture instances of this flow, which is temporarily stable and creates an illusion that organisms are things. Can we speak about the organism at all in such a framework? To show that it is possible we can compare it to a river. It flows and seems to have no clear boundaries, but this does not mean there are no boundaries. There exist multiple ways to set boundaries to a river, ranging from the geographic to the geological and so on. Each one is correct but merely captures a different part of the process–the same is true for organisms. Organisms are processes, and we can put different types of boundaries on them by putting forward different concepts of an organism. Each of these concepts equal, just capturing a slightly different part of this flow of living matter. The sort of boundaries that should be chosen for research will vary from one scientific project to another [83, 85, pp. 69–143].

The above paragraphs show that the pluralistic approach to the concept of an organism can be defended by means of philosophical arguments. Therefore, bioethicists do not have to struggle to develop the universal concept of an organism or to defend monism–of course, if they want to do so, they can. However, it is unnecessary as they can accept pluralism about the concept of the organism with good justification. If they do so, they might choose either option (2) or (3). Given option (2) they might base their investigations on one of the concepts of the organism from biology. Going down this road bioethicists should acknowledge that the merger between all views of organisms, that is the notion of “functional integration,” is nebulous and cannot provide a basis to unequivocally answer whether brain-dead patients are dead or not. Therefore, when they want to base their arguments on the concept of an organism, bioethicists should review the existing concepts and select the one that would be the most appropriate for their arguments. By doing so they assure that their argument will be based on a concept that is rooted in contemporary biological research, rather than conceptualizing their concept which might be less capable of staying in touch with current knowledge and even overlook some important biological aspects.

The option (2) would not be very satisfying for the majority of bioethicists. We think that opening doors for biological ideas can help bioethics by providing the flow of new ideas–and the same is true for biologists. However, it is very likely that a lot of concepts from biology and the philosophy of biology cannot simply be transferred and accepted as they have been developed to serve different research purposes that vary across biological sciences. For instance, people working on development want to understand how species develop from single cells, so they might define organisms along those lines. Evolutionary biologists, in turn, want to understand how populations undergo natural selection, so their concepts might be defined in such a way as to be relevant for this goal. This is far removed from the goals that bioethics pursue. We have presented these concepts to show that even if we stick to the concepts of the organism from biological theories–which should be empirically informed–they are still different about determining death.

The option (3) is an interesting one and seems to be the most appropriate. Going down this road, bioethicists should, just as with the previous option, realize that the merger of “functional integration,” that is common for all understandings of an organism, is nebulous and prone to different yet equally valid operationalizations within the life sciences. Scientists utilize such operationalizations for pragmatic reasons–mainly to accomplish their research goals. Bioethicists engaged in the brain death debate might argue that they also have such goals. In essence, they are theorizing about the biological status of a brain-dead patient. It seems that there are some concepts of an organism, including “bioethical-biological” ones, which implies that the brain-dead patient is alive (e.g., Shewmon’s view), and there are some different perspectives (e.g., Moschella’s master part view) which indicate the opposite–that the brain-dead patient is dead.

Since the strategy for the bioethicists that we are discussing takes organismal pluralism at face value, those who adopt it should not avoid the conclusion that brain-dead patients can be alive given some concepts of an organism, and dead according to equally valid views. This is just the same as the fact that biologists believing in organismal pluralism accept the conclusion that, for instance, protozoans are part of a termite organism (according to the immunological view) and simultaneously are not parts of a termite’s organism (from the zygotic functional perspective). While the acceptance of biological pluralism is not a problem for biologists, with biologists both aware and even calling for it [20], it seems to be a much graver issue for bioethicists. This is because all bioethicists want to investigate the status of brain-dead patients for the sake of providing some objective moral guidance, or at least that which would be invariant within a given society. Most of them believe that it is objectively wrong to cause death. Therefore, they investigate the status of a brain-dead patient for the sake of reaching a conclusion of whether the patient is an entity of the sort that might die at some point [3; 18; 19; 86; 87, pp. 1–520; 88; 89, pp. 89–114]. Others, even though they do not believe that it is always wrong to cause death, uphold that it is objectively wrong not to disclose that a patient has the status of an organism when a decision about the patient’s fate is to be made [38, pp. 1–174; 39; 78; 90].

However, if there is organismal pluralism, as we believe is the case, there is no definitive answer as to whether the brain-dead patient is alive or dead in a biological sense. Therefore, strictly speaking, the information disclosed should be the information about the patient’s organismal status assessed from all biologically valid concepts of an organism. Moreover, bioethicists that believe that causing the biological death of an organism is wrong should accept that there is no definite answer as to whether conduct such as organ removal causes the death of the brain-dead patient. It would be wrong to remove organs from brain-dead patients given some concepts of an organism such as the immunological or Shewmon’s view. It would be morally neutral given some other concepts, such as Moschella’s master part view or the developmental functional view. This second upshot of organismal pluralism is especially problematic from the perspective of ethics, since ethicists and policymakers would like to have a single correct answer when considering whether someone has caused the death of some other human. After all, we cannot both blame and reward someone for a given act.

Another risk coming from the organismal pluralism and from the fact that we conceptualize organisms to pursue some goals is that bioethicists can have different goals in mind. Some of them work on the elaboration of the idea that brain-dead patients are alive and construct a number of arguments for this. As a result, they conceptualize the concept of an organism in a way that fits their philosophical agenda. Others conceptualize organisms differently, leading to a situation in which their concepts imply that brain-dead patients are dead. We do not suggest that they might do so deliberately, but rather this might be done as a by-product of their way of reasoning. Bioethicists rarely conceptualize organisms first and then start thinking about the status of brain-dead patients. Rather, they first develop some sort of intuition (for instance, those driven by their religious views) and then try to develop the concept of the organism as a part of their argumentation. This poses the danger that their views will impinge on how they conceptualize organisms, making the concept biased towards the conclusion they want to develop. This is because the concept of an organism is very elastic and, as we have shown throughout this paper, can be defined in many ways depending on the goals.

The option (4), which might work for some bioethics, is to claim that death cannot be conflated with the cessation of an organism. Perhaps the whole talk about the ‘bioethical-biological’ *concept of an organism* presented in this article springs from some misunderstanding. One could stress that Bernat et al. identified death precisely speaking with “the permanent cessation *of functioning* of the organism *as a whole*” (emphasis added), while the most recent of Bernat’s and Huang’s ideas identify death with cessation of an organism *as a whole*. A similar strategy might also work for the President’s Commission view related to the integrated functioning of the body, or the idea of Nair-Collins that we should identify organism death with the “irreversible cessation of the organismic capacity to maintain homeostasis of the extracellular fluid and thereby resist entropy.” Along these lines, it might be stated that a definition of death cannot be deduced from the sine qua non conditions for the existence of an organism as they appear in theoretical biology. To prove that the death of an organism and its cessation might be distinct phenomena, one could look to paleontological research and argue that permanently non-functioning organisms are dead, yet still exist as organisms, or that the paleontologists’ object of research does not constitute an organism *as a whole,* while at the same time it is still an existent organism. Such a strategy, however, is problematic for several reasons. First, as noted in the introductory section, many bioethicists explicitly or implicitly interpret the debate about the definition of death as precisely a debate about the sine qua non conditions for the existence of an organism. Arguably, for many authors, terms like “permanently disintegrated organism,” an organism that permanently ceased to be a whole, or the “permanently non-functioning organism” strictly mean nothing more than “the former organism” or the entity that used to be an organism but now ceases to be such. Therefore, according to such an approach, a paleontologist would strictly conduct her research on the remains of an organism, not on an organism itself.

Second, although the concept of an organism is quite nebulous and prone to different interpretations, one thing about this notion is more straightforward, namely that organisms are entities that by definition are functionally integrated and constitute wholes that differ from the sum of their parts. This becomes clear if we look at the historical-philosophical analysis of the notion in question provided by Daniel Nicholson:

The concept of organism is grounded in two key organizational relations: (a) the parts–whole relation, according to which an organism is construed as a structurally and functionally differentiated whole, and (b) the inside-outside relation, according to which an organism is construed as an autonomous system capable of maintaining itself in the face of changes to its environment…. The intricate relation between parts and whole was first recognized by Kant in his Critique of the Power of Judgment … in which he observed that living beings are self-organizing systems in the sense that their parts reciprocally produce one another in accordance to the organization of the whole. [52; cf. 91; 92, p. 245/374]

Therefore, again, if one speaks of a permanently functionally disintegrated organism or an organism that no longer constitutes a whole, it is best understood as the “former organism,” the entity that used to be an organism but ceased to be such. So, if death is understood as the functional disintegration of an organism or as a moment when an organism ceases to be a whole, then death is nothing more than the cessation of an organism. Not only could Nicholson’s analysis be recalled here but also the words of Thomas Pradeu, according to whom “asking what a biological individual is [and in particular asking what individual organism is–we would add] means asking what constitutes a countable, relatively well-delineated, and cohesive unit in the living world” [23].

Of course, one could speak about death also in a different biological sense than the cessation of an organism, referring to the opposition between dead and living material. This is one of the most debated issues in biology and the philosophy of biology–one that is especially interesting when scholars try to find out whether viruses are alive [93] or when they try to define life for astrobiological investigations [94]. For both cases, figuring out whether something is living or dead is undoubtedly an important issue to drive research. As Koonin and Starokadomskyy put it, “the ‘dead-alive’ dichotomy in the classification of biological entities seems to present unsolvable conandra whereby the borders of life cannot be clearly defined” [93]. However, in such a sense, it is beyond all controversy that brain-dead patients on artificial support are living since there are living organs, tissues, and cells in their bodies. They belong to the “life” domain of the world. Therefore, in our opinion, the whole discussion about the status of brain-dead patients within the “biological-bioethical” paradigm makes sense if it is a controversy about the existence of some cohesive biological individual such as an organism.

The last problem with the strategy is associated with the ordinary meaning of the phrase “to die.” In everyday talk, it means the cessation of the existence of some living entity. When a person dies, “the person goes out of existence; subsequently, there is no such thing as that person” [95]; (cf. [96; 97, p. 287]), even though the remains of the person might still be present, and even though we might refer to, for example, Socrates by the phrase “dead person,” meaning a person that used to exist at some point. We dare to claim that when we talk about a dead organism, we mean the entity that has gone out of existence as an organism.

## Conclusions

This investigation might lead us to the conclusion that we should accept that there is no single correct answer as to whether a type of human action causes death or not. Perhaps we should assume that there are no definitive answers about moral blame or the prize of performing organ transplantation. However, such a conclusion would be premature. Note that people believed that causing the death of their peers, and especially killing them, was wrong long before the notion of an organism appeared in modern science. They believed this in ancient times even though, strictly speaking, there was no theory of an organism in ancient philosophy, the Bible, the Koran, or the sutras [99]. Perhaps by causing death or killing their peers, people always had in mind something different from causing the cessation of an organism, something of non-reducible moral phenomenology [cf. 8–10, 99].

## Data Availability

Not applicable.

## Footnotes

1 In the United States, Presidential Commission is a special task force ordained by the President to complete specific, special investigation or research. They are often quasi-judicial in nature; that is, they include public or in-camera hearings.
