# Correction to: How many ways can you die? Multiple biological deaths as a consequence of the multiple concepts of an organism

**DOI:** 10.1007/s11017-022-09591-2

**Published:** 2022-10-05

**Authors:** Piotr Grzegorz Nowak, Adrian Stencel

**Affiliations:** Institute of Philosophy, Jagiellonian University, Grodzka 52, 31-044 Kraków, Poland

## Correction to: Theoretical Medicine and Bioethics (2022) 43:127–154 https://doi.org/10.1007/s11017-022-09583-2

The article “How many ways can you die? Multiple biological deaths as a consequence of the multiple concepts of an organism”, was originally published Online First without Open Access. After publication in volume 43, issue 2–3, page 127–154 the author decided to opt for Open Choice and to make the article an Open Access publication. Therefore, the copyright of the article has been changed to © The Author(s) 2022 and the article is forthwith distributed under a Creative Commons Attribution 4.0 International License, which permits use, sharing, adaptation, distribution and reproduction in any medium or format, as long as you give appropriate credit to the original author(s) and the source, provide a link to the Creative Commons licence, and indicate if changes were made. The images or other third party material in this article are included in the article’s Creative Commons licence, unless indicated otherwise in a credit line to the material. If material is not included in the article’s Creative Commons licence and your intended use is not permitted by statutory regulation or exceeds the permitted use, you will need to obtain permission directly from the copyright holder. To view a copy of this licence, visit https://creativecommons.org/licenses/by/4.0.

Also additional funding information has been included in this correction.

The original article has been corrected.

